# Prognostic utility of human complement factor H related protein test (the BTA *stat*® Test)

**DOI:** 10.1054/bjoc.2001.1938

**Published:** 2001-08

**Authors:** M-P Raitanen, E Kaasinen, E Rintala, E Hansson, P Nieminen, R Aine, T L J Tammela, The Finn Bladder Group

**Affiliations:** Departments of Urology and; Pathology, Tampere University Hospital, Box 2000, Tampere, 33521, Finland; Department of Surgery, Hyvinkää Hospital, Hyvinkää, 05850, Finland; Department of Urology, Helsinki University Hospital, Hus, 00029, Finland; Department of Surgery, Vaasa Central Hospital, Vaasa, 65130, Finland; Medical Informatics Group and Department of Psychiatry, University of Oulu Medical School, Oulu, 90220, Finland; Department of Surgery, Seinäjoki Central Hospital and the Finn Bladder Group, Seinäjoki, Finland, 60220

**Keywords:** bladder neoplasms, tumour marker, prognosis

## Abstract

The purpose of the study was to determine, in addition to well-known prognostic factors, histological grade, stage, tumour size and multiplicity, the correlation of BTA *stat* Test on disease free interval (DFI) on primary superficial bladder cancer. A total of 116 patients with newly diagnosed bladder cancer were evaluated in a prospective multicentre study. A voided urine sample was obtained prior to TURB and split for culture, cytology and BTA *stat* testing. Follow-up data for the patients were collected until the first recurrence or the last visit and the DFI was analysed by Kaplan–Meier method and Cox analysis. Ninety-seven of the 116 (83.6%) patients were eligible for analysis. The BTA *stat* Test was positive in 73 (75.3%) patients, whereas cytology detected 20 (20.6%) cases. The DFI was found to be shorter among patients with a positive BTA *stat* Test, and also among those with intermediate or high-grade tumours. The BTA *stat* Test result divided patients with grade 2 tumours into two prognostic groups, in that those testing positive had 68.6% risk of recurrence during the first year compared to 42.9% risk of those with a negative test result (*P* = 0.041). Although the effect of tumour size on DFI was notable, the difference did not reach statistical significance (*P* = 0.064). Number of tumours was not related to DFI, nor was the difference between different stage of tumour of significance. BTA *stat* Test is not only sensitive in detection of primary bladder cancer, but also might have some independent prognostic significance. © 2001 Cancer Research Campaign http://www.bjcancer.com


*British Journal of Cancer* (2001) **85**(4), 552–556
© 2001 Cancer Research Campaign
doi: 10.1054/ bjoc.2001.1938, available online at http://www.idealibrary.com on IDEAL

### Prognostic utility of human complement factor H related protein test (the BTA *stat*® Test)

**M-P Raitanen[1,7], E Kaasinen[3], E Rintala[4], E Hansson[5], P Nieminen[6], R Aine[2], TLJ Tammela[1] and The Finn Bladder Group***

Departments of [1]Urology and [2]Pathology, Tampere University Hospital, Box 2000, 33521 Tampere, Finland; [3]Department of Surgery, Hyvinkää Hospital, 05850 Hyvinkää, Finland; [4]Department of Urology, Helsinki University Hospital, 00029 Hus, Finland; [5]Department of Surgery, Vaasa Central Hospital, 65130 Vaasa, Finland; [6]Medical Informatics Group and Department of Psychiatry, University of Oulu Medical School, 90220 Oulu, Finland, [7]Department of Surgery, Seinäjoki Central Hospital, 60220 Seinäjoki, Finland, and the Finn Bladder Group

**Summary** The purpose of the study was to determine, in addition to well-known prognostic factors, histological grade, stage, tumour size and multiplicity, the correlation of BTA *stat* Test on disease free interval (DFI) on primary superficial bladder cancer. A total of 116 patients with newly diagnosed bladder cancer were evaluated in a prospective multicentre study. A voided urine sample was obtained prior to TURB and split for culture, cytology and BTA *stat* testing. Follow-up data for the patients were collected until the first recurrence or the last visit and the DFI was analysed by Kaplan–Meier method and Cox analysis. Ninety-seven of the 116 (83.6%) patients were eligible for analysis. The BTA *stat* Test was positive in 73 (75.3%) patients, whereas cytology detected 20 (20.6%) cases. The DFI was found to be shorter among patients with a positive BTA *stat* Test, and also among those with intermediate or high-grade tumours. The BTA *stat* Test result divided patients with grade 2 tumours into two prognostic groups, in that those testing positive had 68.6% risk of recurrence during the first year compared to 42.9% risk of those with a negative test result ($P = 0.041$). Although the effect of tumour size on DFI was notable, the difference did not reach statistical significance ($P = 0.064$). Number of tumours was not related to DFI, nor was the difference between different stage of tumour of significance. BTA *stat* Test is not only sensitive in detection of primary bladder cancer, but also might have some independent prognostic significance. © 2001 Cancer Research Campaign http://www.bjcancer.com

**Keywords**: bladder neoplasms; tumour marker; prognosis

Transitional cell carcinoma (TCC) of the bladder is characterized by a heterogeneous group of tumours with a varied malignant potential and natural course. Approximately 80% of the bladder cancers are low-grade superficial tumours (Heney et al, 1983). Although these tumours can be resected transurethrally (TURB), the risk of tumour recurrence is up to 85%, and recurrence often develops during the first year (Heney et al, 1983; Heney et al 1982). Number (Heney et al, 1982; Koch et al, 1986) and size (Heney et al, 1982) of tumours and tumour stage (Heney et al 1982; Kiemeney et al, 1993) and grade (Kurth et al, 1995) have a clear impact on the risk of rapid recurrence. Additionally, early recurrence after TUR has a prognostic effect itself leading to higher risk for recurrences in general (Parmar et al, 1989; Fitzpatrick et al, 1986).

The BTA *stat*® Test (Bion Diagnostic Sciences, Redmond, Washington, USA) is a one-step, rapid immunochromatographic assay that detects a bladder tumour associated antigen in human urine (Sarosdy et al, 1997). The antigen detected by this test has been identified as human complement factor-H related protein (hCFHrp). It has been demonstrated that hCFHrp is produced and secreted by several human bladder cancer cell lines, but not by normal human epithelial keratinocytes (Kinders et al, 1997a, 1997b). It has also been demonstrated by in situ hybridization that bladder tumours produce hCFHrp (Kinders et al, 1998). The function of this protein is similar to that of human complement factor H, i.e. by down-regulating the alternative complement pathway this protein may help bladder cancer cells escape lysis by the host immune system. Thus, it seems possible that expression of hCFHrp, detected by the BTA *stat* Test, could also be used as a prognostic marker.

The BTA *stat* Test has been shown to have equal or superior sensitivity to that of voided urine cytology in detecting primary and recurrent bladder tumours (Sarosdy et al, 1997; Pode et al, 1999; Raitanen et al, 2000), whereas the specificity has been decreased by benign genitourinary condition (Sarosdy et al, 1997) and intravesical BCG treatment (Pode et al, 1999). Although it has also been suggested that false positive BTA *stat* Test in patients under follow-up for TCC might predict tumour recurrence (Sarosdy et al, 1997), the idea of BTA *stat* Test as a prognostic marker on primary bladder tumours has not been introduced or reported earlier. The aim of this study was to evaluate the role of tumour grade, stage, size, and number, and more interestingly, to test our hypothesis that BTA *stat* Test result might be of prognostic importance.

*Received 20 October 2000*
*Revised 30 April 2001*
*Accepted 15 May 2001*

*Correspondence to:* M Raitanen

*Members: M. Ala-Opas and K Tuhkanen, Kuopio University Hospital; T Forssel, Kymenlaakso Central Hospital, Kotka; P. Granbacka and C. Palmberg, Pietarsaari Hospital; T. Liukkonen, Mikkeli Central Hospital; H. Juusela, Hospital of Jorvi, Espoo; H. Korhonen, Satakunta Central Hospital, Pori; P. Hellström and O. Lukkarinen, Oulu University Hospital; M. Nurmi and P. Rajala, Turku University Hospital; J. Permi and V-M. Puolakka, Lappeenranta Central Hospital; E. Rintala, I. Perttilä, Helsinki University Hospital; T. Tammela and M-P. Raitanen, Tampere University Hospital; M. Leppilahti, M. Leskinen. M-P. Raitanen and T. Marttila, Seinäjoki Central Hospital, E. Kaasinen, Hyvinkää Hospital; J. Ervasti, Ähtäri Hospital; A. Mehik, Oulainen Hospital; R. Kauppinen and M. Rauvala, Rovaniemi Central Hospital; J. Viitanen, Pohjois-Karjala Central Hospital, Joensuu; E. Hansson and P. Nylund, Vaasa Central Hospital.

### MATERIALS AND METHODS

This was a prospective, multi-centre study conducted by FinnBladder Group at 18 medical institutions between 1997 and 1999. One hundred and sixteen patients with confirmed primary transitional cell carcinoma of urinary bladder were consecutively recruited in accordance with the Declaration of Helsinki. Tumour had to be confined to the bladder mucosa (Ta) or lamina propria (T1) and curatively treatable by TURB. A total number of 19 patients were excluded from analysis since they were lost to follow-up or were treated with series of intravesical instillations leaving 97 eligible patients, of which 73 (75.3%) were men (mean age 66 years, range 38–88) and 24 (25.0%) were women (mean age 68 years, range 21–86). This study was approved by each local Human Investigations Committee and all the patients gave written informed consent. Five patients (5.2%) have received a single instillation of chemotherapeutic agent immediately after TURP, however, as there were only very few patients with this treatment, those patients were not analysed separately here. Standard follow-up policy was obtained. According to the grade and stage of primary tumour, cystoscopy was performed every 3–6 months for 2 years and if no recurrences were observed during this time the interval was prolonged to 6 –12 months.

Freshly voided urine samples were obtained a minimum of 2 weeks after diagnostic cystoscopy, prior to transurethral resection of tumour (TURB) and split for culture (to exclude urine infection), cytological analysis and BTA *stat* testing. The BTA *stat* Test was performed at each institution by a trained nurse according to the manufacturer's instructions by adding five drops of an untreated voided urine sample into the sample well of the disposable test device using the disposable pipette provided. Five minutes after the addition of urine to the sample well, a qualitative (positive or negative) interpretation was performed. The cytopathological results were analysed by central review (RA) blinded to the results of the cystoscopy, local cytology and the BTA *stat* Test result. Positive voided urinary cytology was defined as Papanicolaou classification 4 and 5. Tumour characteristics such as size and number were recorded. Tumours were graded according to the World Health Organization grading system and staged according to TNM classification at each of the participating hospitals (Mostofi et al, 1973; UICC, 1978).

Primary end-point was time to first recurrence, i.e. the disease-free interval (DFI). The Kaplan–Meier method was used to obtain the estimated DFI curves for the studied variables such as BTA *stat* Test result and the well known prognostic factors (histological grade, stage, tumour size and number). A further stratification was applied to examine the relationship between tumour grade and BTA *stat* Test and their effects on DFI. A number of statistical significance tests have been proposed which compare two Kaplan–Meier curves (Parmar and Machin, 1995). The most widely used testing method, the log-rank test, emphasizes recurrences in the tail of the curves. In our study, we wanted to give more weight to the earlier part of the curve where there are larger number of patients at risk. We also anticipate that BTA *stat* Test may help to detect the particular risk of very short DFI. Therefore, we used Breslow version of the generalized Wilcoxon statistic test, which places more emphasis on the information at the beginning of the DFI curve.

The Cox proportional hazard model (Parmar and Machin, 1995) was used in the multivariate analysis for testing the independence and significance of the effects of the aforementioned variables on recurrence. The model is reported using hazard ratios and their 95% confidence intervals (CI). Hazard ratios demonstrate the relative risk for recurrence. *P*-value of the likelihood ratio test is also reported to confirm the variable as an appropriate prognostic factor. Statistical analysis was performed with SPSS for Windows software (SPSS, 1999).

### RESULTS

The BTA *stat* Test was positive in 73 cases (75.3%) of the 97 patients at the diagnosis of primary bladder carcinoma, whereas only 20 patients (20/86, 23.3%) presented with concurrent positive cytology. The mean follow-up time was 13 months (range 1–48 months, median 9.0). Sixty-six (68.0%) patients had recurrence with the mean time of 8 months (range 1–28 months, median 7). The mean follow-up time in those with no recurrence was 27 months (range 6–48 months, median 28). Distribution of patients according to tumour characteristics, BTA *stat* Test and cytology is summarized in Table 1.

The Kaplan–Meier plot showed that disease-free interval was significantly longer if the patient had negative BTA *stat* Test, as according to the Kaplan–Meier estimates the patients with positive BTA *stat* Test had 63.0% risk of recurrence during the first year of follow-up compared to 39.1% risk of the ones with negative testing ($P = 0.002$, Figure 1A). The difference in DFI between the patients with low-grade and high-grade tumours was also significant, since the patients with grade 1,2 and 3 tumours had 44.2%, 64.6% and 88.9% risks of recurrence during the first year, respectively ($P = 0.0005$, Figure 1B). DFI was not affected by stage of tumour as shown by the quite similar risks of recurrence of 56.0% and 61.0% in patients with Ta and T1 tumours ($P = 0.60$). Although the disease free probability was 53.1% in the patients with single tumour compared to that of 35.2% of patients with multiple tumours, the difference was not statistically significant ($P = 0.284$). Accordingly, no statistically significant distinction could be made either on the basis of tumour size in spite of the consistent trend observed in the disease free estimates, which were at one year 58.8%, 43.2% and 29.7% for tumours smaller than 1 cm, for tumours 1–3 cm, and for tumours larger than 3 cm, respectively ($P = 0.131$). In addition, the impact of tumour size on DFI became more clear if the cut-off point was set on 1 cm, as 41.2% and 61.5% of the patients with tumour size ≤ 1 cm or > 1 cm had

**Table 1** Distribution of patients according to grade, stage, number and size of tumour, and BTA *stat* Test and cytology.

| | Number | (%) | | Number | (%) |
|---|---|---|---|---|---|
| Grade: | | | Size: | | |
| 1 | 46 | (47.4) | < 1 cm | 25 | (25.8) |
| 2 | 42 | (43.3) | < 3 cm | 44 | (45.4) |
| 3 | 9 | (9.3) | > 3 cm | 26 | (26.8) |
| | | | unknown | 2 | (2.1) |
| Stage: | | | | | |
| Ta | 56 | (57.7) | BTA *stat* | | |
| T1 | 36 | (37.1) | Positive | 73 | (75.3) |
| unknown | 5 | (5.3) | Negative | 24 | (24.7) |
| Number: | | | Cytology | | |
| 1 | 62 | (63.9) | 1–3 | 66 | (68.1) |
| 2–3 | 21 | (21.6) | 4–5 | 20 | (20.6) |
| > 4 | 12 | (12.4) | unknown | 11 | (11.3) |
| unknown | 2 | (2.1) | | | |

© 2001 Cancer Research Campaign

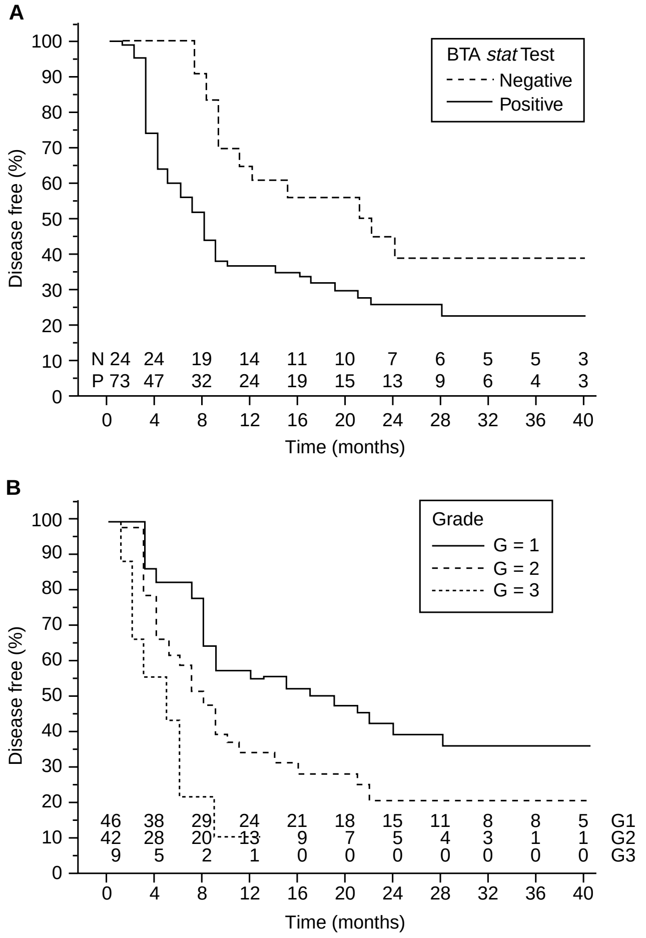

**Figure 1** Kaplan–Meier plot of the disease-free interval of 92 patients according to BTA *stat* Test (**A**) and tumour grade (**B**). The difference in curves is significant (**A**: *P* = 0.002, **B**: *P* = 0.0005). The number of patients at risk at the beginning of each 3-month period is indicated above the time axis

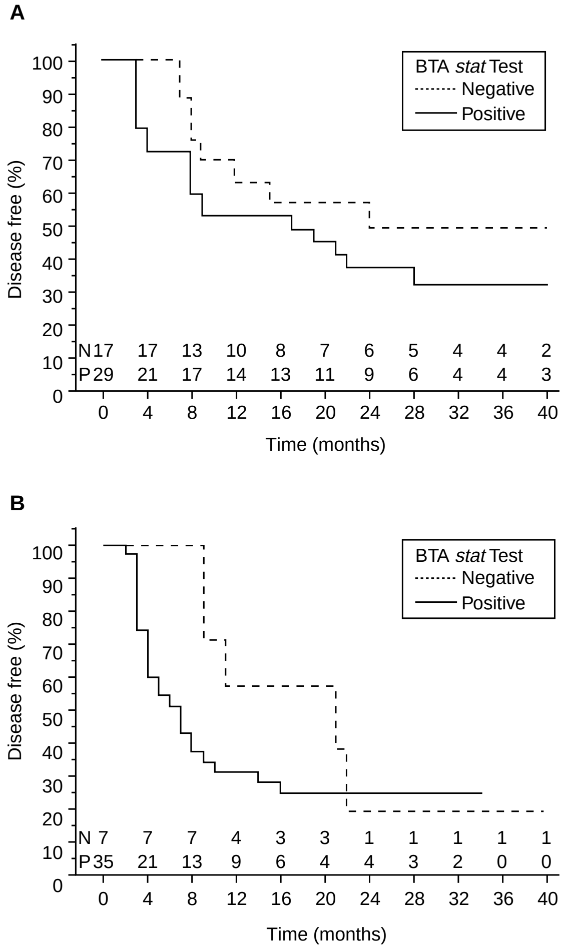

**Figure 2** Kaplan–Meier plot of the disease-free interval of patients according to BTA *stat* Test and tumour grade. (**A**) Patients with grade 1 tumours (*P* = 0.176). (**B**) Patients with grade 2 tumours. The difference in curves in (**B**) is significant (*P* = 0.041). The number of patients at risk at the beginning of each 3-month period is indicated above the time axis. Positive BTA *stat* Test (solid line), negative BTA *stat* Test (dashed line)

recurrence during the first year, respectively (*P* = 0.064).

Although the number of BTA *stat* Test negative patients was low among grade 2 tumours (7/42), the BTA *stat* Test result appeared to divide the patients with grade 2 tumours into two statistically significant prognostic groups (*P* = 0.041, Figure 2B). The risk of recurrence was 68.6% in the patients with positive BTA *stat* Test as compared to 42.9% in the patients with negative results, the latter percentage being quite similar to risk of patients with low grade tumours (44.2%). However, no such statistical distinction according to the BTA *stat* Test result could be made among the low grade tumours (*P* = 0.176, Figure 2A). By contrast, if grade 1 and 2 tumours were analysed as a single group, the difference between BTA *stat* Test positive and negative patients remained significant (*P* = 0.006). All nine patients with grade 3 tumours had positive BTA *stat* Test result, and all but one of them had recurrence. The effect of BTA *stat* Test on DFI was not therefore analysed separately among these patients.

DFI analysis of co-variates was performed according to the Cox model for all the patients for whom complete data on all the variables were available. There was a substantial proportion of patients missing review cytology. In addition, as positive cytology was completely correlated to positive BTA *stat* Test in our earlier study (Raitanen et al, 2000) and BTA *stat* Test was regarded here as an alternative to cytology, cytology was not included in multivariate analysis. Histological grade and BTA *stat* Test result were both independently related to the DFI. Tumour grade implied 1.7-and 3.9-fold risk of recurrence for grade 2 and 3 tumours compared to tumours of low grade. A positive BTA *stat* Test result implied a 2.2-fold risk for recurrence compared to a negative test. In concordance with Kaplan–Meier analyses, the DFI was not affected in the Cox model by size or number of tumours, nor was the tumour stage of prognostic significance (Table 2).

### DISCUSSION

We report here the results of a prospective multicentre study in which the factors effecting DFI were analysed. Ninety-seven eligible patients with primary superficial bladder cancer with mean follow-up time of 13 months were evaluated. We found that, in addition to the well-known prognostic factor predicting early recurrence, tumour grade, also the positive BTA *stat* Test predicted shorter DFI. The independence of the two factors in predicting short-term recurrence was noteworthy, since in our earlier analysis (Raitanen et al, 2000) the sensitivity of BTA *stat* Test was essentially related to the grade of tumour. However, in this study exam-

© 2001 Cancer Research Campaign

**Table 2** Multivariate (Cox model) disease free interval (DFI) analysis of 92 patients with conservatively treated superficial bladder cancer. Hazard ratios (95% CI) and *P* value for additional information in multivariate analysis are given for each covariate

| Variable | Hazard ratio | 95% Confidence interval | *P*-value |
|---|---|---|---|
| BTA *stat* Test | | | 0.02 |
| – negative | 1 | | |
| – positive | 2.2 | 1.1–4.6 | |
| Grade | | | 0.02 |
| – 1 | 1 | | |
| – 2 | 1.7 | 1.0–3.1 | |
| – 3 | 3.9 | 1.5–10.0 | |
| Stage | | | 0.09 |
| – Ta | 1 | | |
| – T1 | 0.6 | 0.3–1.1 | |
| Number | | | 0.98 |
| – Single | 1 | | |
| – multiple | 1.0 | 0.6–1.7 | |
| Size | | | |
| – ≤ 1 cm | 1 | | 0.46 |
| – ≤ 3 cm | 1.0 | 0.5–2.0 | |
| – > 3 cm | 1.5 | 0.7–3.2 | |

ining DFI, the presence of the antigen detected by the BTA *stat* Test showed a potential in distinguishing low and high risk groups among patients with grade 2 tumours.

Superficial bladder cancer includes a heterogeneous group of tumours that vary considerably in histological appearance and malignant potential. The tumour stage (Heney et al, 1982; Kiemeney et al, 1993; Lutzeyer et al, 1982; Malmström et al, 1987) and grade (Kiemeney et al, 1993; Kurth et al, 1995; Lutzeyer et al, 1982) as well as the size (Heney et al, 1982) and number (Heney et al, 1982; Koch et al, 1986; Kiemeney et al, 1993; Lutzeyer et al, 1982) of tumours are well known prognostic factors, in that, multiple, large (> 3 cm), high grade tumours that invade lamina propria (T1) have a shorter disease free interval and more recurrences in general, and more importantly, a higher progression rate and even mortality. Our results confirm the effect of histological grade on the natural course of the disease, as tumour grade was strong predictor for short DFI. However, the impact of number or size of tumours, was not of statistical significance, although differences in DFI were notable. This may be due, at least partly, to the rather small number of patients in the analysis. Moreover, due to the short follow-up time here, evaluating anything but DFI is not justified.

The risk of recurrence is high after TURB (Heney et al, 1982) and although the risk for further recurrences and for progression decreases as the number of years without recurrences increases (Morris et al, 1995), tumour may recur and even progress after five years free of the disease (Thompson et al, 1993). The overall risk of 68.0% for recurrence during the first year is in accordance with previous reports (Heney et al, 1982) and suggests that our prospective study with consecutive patients from 18 centres represents well the population of patients being monitored for conservatively treated superficial bladder cancer. In addition, the mean follow-up time was over 2 years in patients with no recurrence.

In accordance with other studies (Sarosdy et al, 1997; Pode et al, 1999; Raitanen et al, 2000), the sensitivity of BTA *stat* Test was superior to that of cytology, in that BTA *stat* Test and cytology detected 75.3% and 23.3% of tumours, respectively. To date, little has been reported about the prognostic effect of BTA *stat* Test. It has been suggested that it may be possible that the BTA *stat* Test predicts tumour recurrence (Pode et al, 1999). It has also been reported that positive BTA *stat* Test in a patient with negative cystoscopy may predict oncoming recurrence. Sarosdy et al reported that in their study of 107 patients under follow-up for bladder cancer but with no sign of recurrence at cystoscopy, 32 (29.9%) had false positive BTA stat Test, of which 31 % developed recurrence between 3 and 12 months later (Sarosdy et al, 1997). It is noteworthy that in their study, the current status of the disease on these patients was evaluated only on the basis of cystoscopy.

The antigen detected by the BTA *stat* Test has been identified as human complement factor-H related protein (hCFHrp), a varient of human complement factor H (FH) by partial amino acid sequence analysis. The composition, structure, and function of hCFHrp are similar to those of human complement FH and studies have shown that expression of proteins with CFH-like activities may confer selective growth advantage to cancer cells in vivo by decreasing complement activity, thus allowing the cancer cells to escape lysis by the host immune system (Kinders et al, 1997a; 1997b; 1998).

FH, a soluble negative regulator of the complement system, is produced and secreted by most TCCs of the bladder (Kinders et al, 1998). In situ hybridization experiments have shown that bladder tumours also produce FH messager RNA (mRNA), while normal bladder epithelium produces little or no mRNA (Corey et al, 1998). Although the significance of this phenomenon with regard to both biochemistry and cancer biology remains to be established, the data available suggest that factor H plays an important role in tumour survival (Kinders et al, 1997a, 1997b, 1998). So it can be suggested that when escaping lysis by the immune system the seeded cells during TURB or small residual tumours will survive more often and thus develop recurrency faster than those not secreting factor H. It can also be speculated that positive BTA *stat* Test might be associated with tumourgenesis of bladder epithelium and therefore possibly predict development of a new tumour in another place. These speculations will, however, be the topic of further research.

Unfortunately our short follow-up time does not allow discussion of the long-term prognostic effect of these studied factors. However, these findings confirm the earlier reports of histological grade as a prognostic marker affecting DFI. More importantly, our results suggest that expression of human complement factor H related protein detected by BTA *stat* Test could be used for determining follow-up policy in some cases. Compared to other prognostic factors, the presence of the BTA antigen may have two utilities: detection of new tumours and prediction of a shorter DFI for tumours expressing hCFHrp.

As interesting as the prognostic dimension of the BTA *stat* Test is, there are some aspects that must be kept in mind as the clinical usability of this test is discussed. A majority (75.3% here) of the patients with primary bladder cancer are BTA *stat* positive, predicting shorter DFI, and the remaining 24.7% with a negative test predict better outcome. However, a test detecting the smaller group of patients with poor outcome would be more useful in clinical practice. Additionally, as a negative BTA *stat* Test predicts better outcome, it seems possible to decrease the number of cystoscopies by prolonging the interval in these patients. The safest group of patients would be those with grade 1 tumour, however, we failed to prove any significant difference in DFI by BTA *stat* Test result in this group. Furthermore, as all high grade tumours tested positive, the prognostic use of BTA *stat* Test is not justified

*© 2001 Cancer Research Campaign*

in these tumours. By contrast and more interestingly, the decreased risk (42.9% vs 68.6%) for short-term recurrence among patients with grade 2 tumours testing negative could have clinical implications, although the majority (83.3%) of the patients with grade 2 tumours tested positive. In addition, a similar statistically significant distinction could be made on the basis of the negative test result if grade 1 and 2 tumours were grouped and analysed together. There appears to be a relationship between grade and BTA *stat* Test result according to our results; while negative BTA *stat* Test implies decreased risk of recurrence irrespective of grade, the positive test indicates increased risk of recurrence, the magnitude of which is determined by grade.

We found that the BTA *stat* Test is not only sensitive for the diagnosis of primary bladder cancer but also has prognostic utility in patients with negative results. Although the clinical importance of the observed independent prognostic dimension of the BTA *stat* Test remains somewhat obscure, our data further underlines the role of BTA *stat* Test as a more potent diagnostic tool (in most cases) than cytology in the diagnosis of bladder cancer. The BTA *stat* Test also helped to differentiate high grade and low risk subgroups for short DFI in patients with grade 2 tumours. Therefore, it might be suggested that BTA *stat* Test could replace routine cytology in the diagnosis of bladder cancer, and possibly, could also be used as a tool together with other prognostic markers in determining patients follow-up policy. The possible contribution of BTA *stat* Test should be viewed in the current treatment context, where predictability of the course of the disease remains unsatisfactory. In addition, there is an urgent need for reduction of the large number of negative follow-up cystoscopies, which are costly and produce patient discomfort. One possibility could be in increasing the interval between follow-up cystoscopies in those having negative test result. However, prospective trials are needed to confirm the utility of BTA *stat* Test in these connections.

### ACKNOWLEDGEMENTS

This study was supported by the Medical Research Fund of Tampere University.

### REFERENCES

Corey MJ, Kinders RJ, Brown L, Rowley H and Vessela R (1998) Factor H related proteins are upregulated in bladder cancer. *Proc Am Assn Cancer Res* **39**: 263 (abstr)

Fitzpatrick JM, West AB and Butler MR (1986) Superficial bladder tumours (stage pTa, grades 1 and 2): The importance of recurrence pattern following initial resection. *J Urol* **135**: 920–922

Heney NM, Nocks BN, Daly JJ, Prout GR Jr, Newall JB, Griffin BB, Perrone TL and Szyfelbein WA (1982) Ta and T1 bladder cancer: Location, recurrence and progression. *Br J Urol* **54**: 152–157

Heney NM, Ahmed S, Flanagan MJ, Frable W, Corder MP, Hafermann M and Hawkins IR (1983) Superficial bladder cancer: progression and recurrence. *J Urol* **130**: 1083–1086

Kiemeney LALM, Witjes JA, Heijbroek RP, Verbeek ALM and Debruyne FMJ (1993) Predictability of recurrent and progressive disease in individual patients with primary superficial bladder cancer. *J Urol* **150**: 60–64

Kinders R, Root R, Jones J, Bruce C and Hass M (1997a) Complement factor H-related proteins are expressed in bladder cancer. *Proceedings of the Eighty–Eighth Annual Meeting of American Association for Cancer Research, San Diego, CA USA*, **38**: 29, Abstract No. 189

Kinders R, Jones J, Root R, Murchison H, Bruce C, Williams L and Hass M (1997b) Human bladder Tumour antigen is a member of the RCA (regulators of complement activation) gene family. *J Urol* suppl 4: **157**: 28, Abstract no. 110

Kinders R, Jones T, Root R, Bruce C, Murchison H, Corey M, Williams L, Enfield D and Hass GM (1998) Complement Factor H or a related protein is a marker for transitional cell cancer of the bladder. *Clinical Cancer Research*, Oct., **4**: 2511–2520

Koch M, Hill GB and McPhee S (1986) Factors affecting recurrence rates in superficial bladder cancer. *J Natl Cancer Inst* **76**: 1025–1029

Kurth KH, Denis L, Bouffioux CH, Sylvester R, Debruyne FM, Pavone–Macaluso M and Oosterlink W (1995) Factors effecting recurrence and progression in superficial bladder tumours. *Eur J Cancer* **31A**: 1840–1846

Lutzeyer W, Rübben H and Dahm H (1982) Prognostic parameters in superficial bladder cancer: and analysis of 315 cases. *J Urol* **127**: 250–252

Malmström P–U, Busch C and Norlén BJ (1987) Recurrence, progression and survival in bladder cancer. *Scand J Urol Nephrol* **21**: 185–195

Morris SB, Gordon EM, Shearer RJ and Woodhouse CRJ (1995) Superficial bladder cancer: for how long should a tumour-free patient have check cystoscopies? *Br J Urol* **75**: 193–196

Mostofi FK, Sobin LH and Torloni H (1973) *Histological typing of urinary bladder tumours*. World Health Organization: Geneva

Parmar MKB, Freedman LS, Hargreave TB and Tolley DA (1989) Prognostic factors for recurrence and follow-up policies in the treatment of superficial bladder cancer: report from the British Medical Research Council Subgroup on Superficial Bladder Cancer. *J Urol* **142**: 284–288

Parmar MKB and Machin D (1995) *Survival analysis. A practical approach*. John Wiley Chichester

Pode D, Shapiro A, Wald M, Nativ O, Laufer M, Kaver I (1999) Noninvasive detection of bladder cancer with the BTA stat test. *J Urol* **161**: 443–446

Raitanen M-P and Tammela TLJ (1995) Impact of tumour grade, stage, number and size, smoking and sex on the recurrence rate and disease-free interval in patients with transitional cell carcinoma of the bladder. *Ann Chir et Gyn* **84**: 37–41

Raitanen M-P, Marttila T, Kaasinen E, Rintala E, Aine R and Tammela TLJ (2000) Sensitivity of human complement factor H related protein (the BTA *stat* Test) test and voided urine cytology in the diagnosis of bladder cancer. *J Urol* **163**: 1689–1692.

Sarosdy M, Hudson M, Ellis WJ, Soloway MS, deVere White R, Sheinfeld J, Jarowenko MV, Schelhammer PF, Schervish EW, Patel JV, Chodak GW, Lamm DL, Johnson RD, Henderson M, Adams G, Blumenstein BA, Thoelke KR, Pfalzgraf RD, Murchison HA and Brunelle SL (1997) Improved detection of recurrent bladder cancer using the Bard BTA stat Test. *Urology* **50**: 349–353

SPSS (1999) *Advanced Models 9.0*. SPSS Inc: Chigaco

Thompson RA Jr, Campbell EW Jr and Kramer HC (1993) Late invasive recurrence despite long-term surveillance for superficial bladder cancer. *J Urol* **149**: 1010–1011

Union Internationale Contre le Cancer (1978) *TNM Classification of Malignant Tumours*. 3rd edn International Union Against Cancer: Geneva

 *© 2001 Cancer Research Campaign*
